# Behavioral Sexual Dimorphism in School-Age Children and Early Developmental Exposure to Dioxins and PCBs: A Follow-Up Study of the Duisburg Cohort

**DOI:** 10.1289/ehp.1306533

**Published:** 2013-11-22

**Authors:** Gerhard Winneke, Ulrich Ranft, Jürgen Wittsiepe, Monika Kasper-Sonnenberg, Peter Fürst, Ursula Krämer, Gabriele Seitner, Michael Wilhelm

**Affiliations:** 1 Medical Institute of Environmental Hygiene at Heinrich-Heine-University (HHU) Düsseldorf, Düsseldorf, Germany (retired); 2 IUF-Leibniz Research Institute of Environmental Medicine, Düsseldorf, Germany; 3 Department of Hygiene, Social and Environmental Medicine, Ruhr-University Bochum, Bochum, Germany; 4 Chemical and Veterinary Analytical Institute Münsterland-Emscher-Lippe, Münster, Germany

## Abstract

Background: Polychlorinated dibenzo-*p*-dioxins and dibenzofurans (PCDD/Fs) and polychlorinated biphenyls (PCBs) are persistent organic pollutants that have been characterized as endocrine-disrupting chemicals (EDCs).

Objectives: Within the Duisburg birth cohort study, we studied associations of prenatal exposure to PCDD/Fs and PCBs with parent-reported sexually dimorphic behavior in children.

Methods: We measured lipid-based and WHO_2005_-TEQ (toxic equivalents established in 2005 by the World Health Organization)–standardized PCDD/Fs and PCBs in maternal blood samples and in early breast milk using gas chromatography/high-resolution mass spectrometry. At the child’s age of 6–8 years, parents (mostly mothers) reported sex-typical characteristics, preferred toys, and play activities using the Pre-School Activities Inventory (PSAI), which was used to derive feminine, masculine, and difference (feminine – masculine) scores. We estimated exposure–outcome associations using multivariate linear regression. A total of 91–109 children were included in this follow-up.

Results: Mean blood levels of summed WHO_2005_-TEQ–standardized dioxins (ΣPCDD/Fs) were 14.5 ± 6.4 pg/g blood lipids, and ΣPCBs were 6.9 ± 3.8 pg/g blood lipids, with similar values for milk lipids. Regression analyses revealed some highly significant interactions between sex and exposure—such as for ΣPCBs in milk, pronounced positive (boys: β = 3.24; CI = 1.35, 5.14) or negative (girls: β = –3.59; CI = –1.10, –6.08) associations with reported femininity. Less pronounced and mostly insignificant but consistent associations were found for the masculinity score, positive for boys and negative for girls.

Conclusions: Given our results and the findings of previous studies, we conclude that there is sufficient evidence that these EDCs modify behavioral sexual dimorphism in children, presumably by interacting with the hypothalamic–pituitary–gonadal axis.

Citation: Winneke G, Ranft U, Wittsiepe J, Kasper-Sonnenberg M, Fürst P, Krämer U, Seitner G, Wilhelm M. 2014. Behavioral sexual dimorphism in school-age children and early developmental exposure to dioxins and PCBs: a follow-up study of the Duisburg Cohort. Environ Health Perspect 122:292–298; http://dx.doi.org/10.1289/ehp.1306533

## Introduction

Polychlorinated dibenzo-*p*-dioxins and dibenzofurans (PCDD/Fs), also referred to as dioxins, and polychlorinated biphenyls (PCBs) are persistent organic pollutants (POPs). Because of their accumulation in the food chain and their possible toxicity at even environmental background concentrations, they are still of environmental concern, although exposures have markedly decreased in the general population (Wittsiepe et al. 2000). Dioxins and PCBs are mixtures of congeners that differ in the number and the position of chlorine atoms on the two ring systems. Depending on the degree of chlorination, their biological half-life varies from days to years (Ogura 2004). PCDD/Fs are by-products of combustion processes. PCBs were synthesized and used as synthetic oils in many diverse industrial applications from 1929 until their production and further use was discontinued in the late 1970s.

A broad spectrum of toxicological properties has been reported for both PCDD/Fs and PCBs including neurodevelopmental dysfunction (Schöler und Färber 2002). A more recent emphasis is on their established interference with endocrine systems, such as the hypothalamic–pituitary–thyroid (HPT) and the hypothalamic–pituitary–gonadal (HPG) axes, which also play a regulatory role in brain development (Bergman et al. 2013). Vreugdenhil et al. (2002) reported evidence suggesting that gender-related play behavior as rated by the mothers by means of the Pre-school Activities Inventory (PSAI) (Golombok and Rust 1993) was modified in an antiandrogenic and estrogenic fashion by pre-/perinatal levels of PCBs and PCDD/Fs in a cohort of 158 Dutch children. This was the first epidemiological report to suggest that endocrine disruptors (EDCs) may alter the normal sexual structuring of the brain with resulting behavioral sequelae. In a more recent study, and again using the PSAI, similar modification of sex-related behavior was reported after prenatal exposure to phthalates, another group of widespread environmental EDCs (Swan et al. 2010).

Thyroid as well as steroid hormones, namely androgens and estrogens, are known to play a crucial role in brain development (Weiss 2011). Thyroid hormones are important in orchestrating basic neurobiological processes such as proliferation, differentiation, and migration of neurons as well as the differentiation of glial cells. Gonadal hormones are involved in forming the sexually dimorphic brain. Governed by genetic programming, the initially undifferentiated primate fetus is turned into a male structure after the testis is formed and testosterone is excreted. In humans and other primates testosterone is converted by the enzyme 5-α-reductase to dihydrotestosterone (DHT), which is the proximal metabolite responsible for the masculinization of the external genitalia and the brain (Weiss 2011).

In dealing with dioxins and PCBs as EDCs in the environment, we must consider the complexity of mixtures of individual congeners with presumably different endocrine activities. In the case of PCBs, parent congeners as well as their metabolites, particularly the hydroxylated species (OHCBs), must be taken into account when trying to evaluate possible adverse effects of environmental exposure. Different *in vitro* bioassays have shown OHCBs to have antiestrogenic properties (Kramer et al. 1997; Moore et al. 1997), whereas both estrogenic (increased uterus weight) and antiestrogenic effects were observed following intraperitoneal injections of two PCB congeners (52 and 77) or a technical PCB mixture (Arochlor 1242) into immature rats (Jansen et al. 1993).

Taken together, these experimental and epidemiological observations suggest that *in utero* exposure to PCBs, and perhaps to PCDD/Fs, may interfere with endocrine systems during development and particularly with the functioning of the HPG axis, thus modifying sexually dimorphic brain development with subsequent permanent behavioral alterations in boys and girls. To replicate the Dutch study and clarify several uncertainties in this field, we conducted a follow-up study of the Duisburg birth cohort at school age.

We recruited the Duisburg birth cohort between 2000 and 2002. Contrary to previous observations in another cohort with higher levels of PCB exposure (Walkowiak et al. 2001), we found no evidence of PCB-related neurodevelopmental delay or deficits in this cohort up to 24 months of age (Wilhelm et al. 2008a). However, concentrations of gonadal hormones in cord serum, namely testosterone in girls and estradiol in boys, varied in association with PCDD/Fs and PCBs measured in maternal blood during pregnancy and in maternal milk (Cao et al. 2008). Furthermore, serum dehydroandrosterone sulfate concentrations were positively associated with dioxins and PCBs in Duisburg cohort children at 6 and 8 years of age (Rennert et al. 2012).

The aim of the present follow-up of the Duisburg birth cohort was to study associations between prenatal exposure to dioxins and PCBs at the current low levels of environmental exposure and behavioral sexual dimorphism.

## Materials and Methods

The study protocol of both the parent and the follow-up studies was approved by the Ethics Committee of the Medical Faculty of Ruhr-University Bochum (registry no. 3220-08 and 3221-08), and both study elements were conducted in accordance with the ethical principles for medical research involving human subjects as defined in the Declaration of Helsinki. All assessments were performed after written informed parental consent in both parts of the Duisburg birth cohort study.

*Study area*. The study was conducted in Duisburg, Germany. Duisburg is located on the Rhine River and belongs to the Ruhr District, formerly an important agglomeration area in Germany for heavy industries. Further details have been given elsewhere (Wilhelm et al. 2007).

*Participants*. We recruited 232 pregnant women 18–42 years of age living within a predefined area from September 2000 through October 2002. Healthy mother–infant pairs were eligible for final analysis if they spoke German or Turkish, had ≤ 3 children (including the index child), had no serious complications or illnesses during pregnancy or parturition, and their child was born at term (weeks 38–42 of pregnancy) with an APGAR score of at least 8, and without congenital anomalies (Wilhelm et al. 2008a). For the follow-up study, we contacted the parents again and asked them to complete the accompanying PSAI. One hundred thirty-four parents or mothers (57.8%) returned the completed PSAI, with 121 of them fulfilling the criteria of eligibility.

*Collection of biological material*. Maternal blood (50 mL) was taken during weeks 28–43 of gestation (median, week 32). We collected maternal milk from nursing mothers during the first 3 weeks after parturition using collection pumps (self administration). The mothers were asked to collect a total of about 150 mL of milk before or after nursing within 4–5 days. Collected milk was first kept in a 250-mL bottle in a home refrigerator until transportation for final storage at –20°C until analysis. For further details, see Wilhelm et al. (2008a).

*Analysis of biological material*. Blood samples were analyzed for PCDD/Fs and PCBs at the Department of Hygiene of the Ruhr-University Bochum, and milk samples at the Chemical and Veterinary State Control Laboratory, Münster, Germany. Both laboratories have successfully participated in regular quality assurance exercises for PCBs organized by the Deutsche Gesellschaft für Arbeitsmedizin und Umweltmedizin (German Society for Occupational and Environmental Medicine; Munich, Germany). To our knowledge, no comparable regular efforts exist for PCDD/Fs in Germany. The analytical procedures for measuring PCDD/Fs and PCBs have been described in detail elsewhere (Wittsiepe et al. 2007). Final measurements were done by means of combined high-resolution gas chromatography/high-resolution mass spectrometry, and nondetectable concentrations were taken as 0.5 of the limit of detection (LOD). For the determination of PCDD/Fs in blood, the standard deviation of the method was < 10% for most congeners and up to 25% near the LOD. Recovery rates were 70–95%, and the LODs for TCDD/Fs in blood were < 1 pg/g lipid. Similar values held for determinations in milk. We used laboratory blanks and quality controls to check for reliability. High correlations between levels of POP concentrations in milk and blood (see Supplemental Material, Table S1), measured in two independent laboratories, may be taken as measures of adequate internal quality control for both PCBs and PCDD/Fs. Lipid-adjusted concentrations are given, with lipids measured gravimetrically. We also measured lead and cadmium in full blood, and mercury in urine, but did not account for these exposures in the present analysis.

*Exposure assessment*. We measured a broad spectrum of polychlorinated POPs, namely PCDD/Fs and PCBs both in maternal serum during pregnancy and in early maternal milk samples: 17 PCDD/Fs and 18 PCBs (see Supplemental Material, Table S2). They were lipid based and combined into eight conventional WHO_2005_-TEQ–weighted (toxic eqivalents established in 2005 by the World Health Organization) summary measures (van den Berg et al. 2006): ΣPCDD/F, Σnon-*ortho* PCBs, Σmono-*ortho* PCBs, ΣPCBs, ΣPCDD/F + PCB, ΣPCDD/F, the NATO/CCMS (North Atlantic Treaty Organization Committee on the 33 Challenges of Modern Society)–TEQ–weighted ΣPCDD/Fs, and the unweighted total of six marker PCBs (28, 52, 101, 138, 153, 180).

As expected, most of these summary measures are highly correlated, typically in excess of *r* = 0.75, both within and across matrices (see Supplemental Material, Table S1). In accordance with a recent publication from the Duisburg birth cohort (Rennert et al. 2012) and to keep the list of exposure variables reasonably short, we excluded the non-*ortho*- and the mono-*ortho*-substituted PCBs, the unweighted Σ6-PCBs, and the NATO/CCMS-TEQ–weighted dioxins from further consideration. Thus, the WHO_2005_-TEQ–weighted ΣPCDD/Fs and PCBs and the WHO_2005_-TEQ–weighted ΣPCDD/F + PCB remained as relevant complex indices of POP exposure in the list of independent variables of exposure.

*Assessment of sexually dimorphic behavior*. Childhood play typically shows pronounced behavioral sex differences (Collaer and Hines 1995). Following the previous Dutch study by Vreugdenhil et al. (2002) and in accordance with a study reporting an association between reduced masculine play and prenatal phthalate exposure (Swan et al. 2010), we used the PSAI (Golombok and Rust 1993), to be completed by German and German speaking non-German parents. The PSAI consists of 24 items grouped into three categories: preferred toys (e.g., guns, dolls), preferred activities (e.g., playing house, fighting), and behavioral characteristics (e.g., enjoys rough and tumble play, likes pretty things). For each of these items the perceived frequency of occurrence during the preceding month (never, hardly ever, sometimes, often, very often) was reported by the parents (mostly mothers). Like Vreugdenhil et al. (2002), we calculated a masculine score (the sum of boy-typical frequency-weighted items), a feminine score (same for girl-typical items), and a difference score (feminine score – masculine score). Some psychometric properties of the PSAI in terms of split-half reliabilities (*r* = 0.88) and validity (mothers’ vs. teachers’ ratings: *r* = 0.48 for girls and *r* = 0.37 for boys) have been established (Golombok and Rust 1993).

*Statistical analysis*. We used SAS version 9.01 (SAS Institute Inc., Cary, NC, USA) for statistical analyses. PCDD/F and PCB exposure levels (WHO_2005_-TEQ weighted) in blood and milk samples of the mother were used as proxy measures of prenatal exposure. We estimated associations between prenatal exposure and the different PSAI measures by multiple linear regression analysis.

*A priori* we selected a comprehensive list of potential confounders/covariates for adjustment. This initial list included the following variables: parental education (low/medium/high; highest of either father or mother), German nationality (no/yes), siblings (older or younger as no/yes separately), quality of the home environment [continuous HOME (Home Observation for Measurement of the Environment) score (Caldwell and Bradley 1984)], alcohol use (no/yes) or smoking (no/yes) during pregnancy, duration of pregnancy (weeks), birth weight (grams), age of the child (years), body mass index (BMI) of the child (kilograms per meter squared), sex of child (female/male), maternal IQ (vocabulary score), and total maternal milk intake via nursing (liters).

The variables quality of the home environment as given by the HOME score, maternal IQ, and total maternal milk intake via nursing need some explanation. The HOME inventory (Caldwell and Bradley 1984) is based on a structured interview of an external observer covering aspects of the quality of mother–child interaction in the home environment. This measure has successfully been introduced in cohort studies on the effects of environmental lead on cognitive development (e.g., Lanphear et al. 2005), and was also used as a covariate in neurodevopmental PCB studies (e.g., Walkowiak et al. 2001). Maternal IQ is typically included in regression modeling of developmental studies on the effects of neurotoxic contaminants such as lead or PCBs to account for hereditary modification (Winneke 2011). In the present study, we selected the vocabulary subtest from the German Wechsler Adult Intelligence Scale (WAIS) (Wechsler Intelligenztest für Erwachsene; Tewes et al. 2006) as a short substitute measure for Full-Scale maternal IQ. Furthermore, total maternal milk intake was determined as follows: Using questionnaire-based retrospective information of the mothers on the relative contributions of maternal milk to the monthly infant diet, and assuming an average daily drinking volume for a fully nursed baby of 800 g, we estimated a total postnatal maternal milk intake via nursing.

Variables from the predefined set of potential covariates were included in the final regression model if they were correlated with one of the PSAI scores (*p* < 0.2) when adjusted for one of the exposure variables and the other potential confounders. The HOME score and BMI did not meet this criterion. Furthermore, because parental education and maternal IQ were highly correlated (Spearman *r* = 0.51; *p* < 0.001), and model coefficients for the exposures did not differ substantially when adjusted for one variable versus both variables, we included the continuous variable maternal IQ, but not the categorical variable parental education, in the final model.

We expressed the results of the regression analyses for the association between POP exposure and the PSAI measures as adjusted regression coefficients (β) together with their 95% CIs and Wald *p*-values. Associations were estimated using separate models for girls and boys. We derived *p*-values for effect modification by sex based on Wald *p*-values for product interaction terms (sex × exposure) from models that included the same covariates plus lower-order terms for sex and exposure. To be consistent with previous work (Vreugdenhil et al. 2002; Walkowiak et al. 2001), we used PCDD/F and PCB exposure variables after log_2_ transformation; thus, their adjusted regression coefficients estimate changes of the PSAI measures associated with any doubling of the exposure. Because regression analyses of the total study group (boys and girls) were applied to estimate the interaction between exposure and sex, the *p*-values for only the interaction terms are given in “Results.”

## Results

*Sample*. Characteristics of this mother–child cohort available for follow-up are given in Table 1. Children, on average, were < 7 years of age (6.6 years), and half of them were boys (50.4%). Compared with the original sample of 2002 (Wilhelm et al. 2008a), the proportion of non-Turkish families remained practically unchanged (88.4% vs. 88.9%); however, loss to follow-up resulted in a better educated (66.1% vs. 56.6% for highest level) and a slightly more health conscious mother–child sample in terms of nonsmoking (79.3% vs. 77.3%) during pregnancy.

**Table 1:** Description of the follow-up sample with size (*n* = 121).

Characteristic	Mean ± SD or *n* (%)
Mothers	
Age at parturition (years)	32.0 ± 4.6
Level of education	
Low (primary school)	9 (7.4)
Medium (secondary school)	32 (26.5)
High (gymnasium)	80 (66.1)
Nationality (German)	107 (88.4)
Length of gestation (weeks)	39.8 ± 1.0
Smoking during pregnancy (yes)	25 (20.7)
Alcohol consumption during pregnancy (yes)	16 (13.2)
Quality of the home environment (HOME score)^*a*^	42.9 ± 1.8
Maternal IQ (Vocabulary score from WAIS)^*b*^	11.1 ± 2.8
Children
Age at completion of PSAI (years)	6.6 ± 0.5
Sex (male)	61 (50.4)
Birth weight (kg)	3.5 ± 0.5
Breastfeeding (yes)	110 (90.9)
Total maternal milk intake via nursing (L)	133.7 ± 86.1
Younger siblings (yes)	48 (39.7)
Older siblings (yes)	58 (47.9)
BMI at completion of PSAI (kg/m^ 2^)	16.0 ± 1.7
^***a***^Some missing values, *n* = 115. ^***b***^Some missing values, *n* = 112.	

The average HOME score of 42.9 in the present study population is slightly higher than that previously found in the Düsseldorf study (41.0; Walkowiak et al. 2001). The mean (± SD) value of the vocabulary test as a proxy for maternal IQ (11.1 ± 2.8) was slightly higher than expected based on the German normative IQ sample (10.0 ± 3.0).

*Exposure.* The measured concentrations of the different exposure complexes for PCBs and dioxins in terms of different distribution characteristics are listed in Table 2; we also give the corresponding values for the complete exposure matrix (see Supplemental Material, Table S3). Most of the values in Table 2 are considerably lower than those reported in other studies (see “Discussion”).

**Table 2:** Prenatal exposure to polychlorinated POPs in maternal blood and milk and total postnatal uptake of maternal milk fat via nursing^*a*^ in the Duisburg birth cohort.

Polychlorinated POPs	*n*	Mean ± SD	Minimum	P5	P50	P95	Maximum
PCDD/F (pg/g blood lipid)	118	14.5 ± 6.4	2.9	6.0	13.0	28.5	32.9
PCB (pg/g blood lipid)	118	6.9 ± 3.8	1.2	2.0	6.0	13.7	25.5
PCDD/F + PCB (pg/g blood lipid)	118	21.5 ± 9.6	4.2	8.0	19.5	38.9	58.4
PCDD/F (pg/g milk lipid)	101	11.6 ± 5.0	3.0	4.5	10.9	19.8	29.4
PCB (pg/g milk lipid)	101	9.0 ± 4.9	1.9	3.3	8.6	16.2	32.2
PCDD/F + PCB (pg/g milk lipid)	101	20.5 ± 9.1	5.0	7.9	19.8	35.8	50.8
P5, P50, and P95 are 5th, 50th, and 95th percentiles. ^***a***^WHO_2005_-TEQ–weighted sum.							

*Outcome.* The three outcome measures of the PSAI as used by Vreugdenhil et al. (2002) are covered in Table 3 for boys and girls. For two female participants, the masculinity section of the PSAI was incomplete.

**Table 3:** PSAI scores for boys and girls.

Score	*n*	Mean ± SD	Minimum	P5	P50	P95	Maximum
Girls
Femininity	60	28.2 ± 6.9	13.0	16.5	28.5	39.0	43.0
Masculinity^*a*^	58	17.6 ± 4.8	8.0	8.0	17.5	28.0	32.0
Difference	58	10.7 ± 7.6	–6.0	–2.0	10.5	24.0	31.0
Boys
Femininity	61	11.2 ± 4.5	2.0	4.0	11.0	20.0	22.0
Masculinity	61	28.3 ± 6.0	17.0	19.0	28.0	37.0	45.0
Difference	61	–17.1 ± 7.3	–32.0	–26.0	–17.0	–4.0	0.0
P5, P50, and P95 are 5th, 50th, and 95th percentiles. ^***a***^Missing masculinity scores for two girls.							

The basic individual measures underlying Table 3 are the frequency-weighted (1 = never to 5 = very often) sum of masculine items and sum of feminine items. The assumption underlying the PSAI is that boys and girls do present with some characteristics of the opposite sex. The mean differences between boys and girls for the respective score values are pronounced and highly significant (*p* < 0.0001). This is also true for the difference score, which, following Vreugdenhil et al. (2002), was calculated as feminine score – masculine score. This supports the validity of the translated PSAI for this age group, which, on average, is slightly above preschool age (Table 1). For comparison, we have also calculated the original composite scores of Golombok and Rust (1993): composite score = 48.25 + 1.1 × difference score. We found (mean ± SD) 67.1 ± 8.0 for boys and 36.5 ± 8.4 for girls; these are close to the population-based mean scores of the PSAI authors: 64.2 ± 8.8 for boys and 35.1 ± 9.4 for girls (Golombok et al. 2008).

*Regression analyses*. The final regression models included as covariates German/non-German nationality, older siblings, younger siblings, alcohol and smoking during pregnancy, duration of pregnancy, birth weight, age of the child, maternal IQ, and total maternal milk intake via nursing. Furthermore, sex and the product interaction terms (sex × exposure) were also included if effect modification by sex was to be estimated. Because of missing values for the covariate maternal IQ, the available sample sizes in the regression analyses were reduced to *n* = 109 and *n* = 92, respectively, when using maternal blood or maternal milk as matrix of the prenatal exposure measures, and were downsized additionally by one female case for the masculine score.

As shown in Table 4, exposures based on concentrations in maternal blood were not significantly associated with PSAI scores of “masculinity” for both boys and girls, whereas levels of PCDD/Fs and PCDDs/Fs + PCBs in breast milk were significantly associated among girls. However, for both matrices of the prenatal exposure measures, we observed a consistent overall pattern of positive associations of POP exposures with the masculine score among boys and of negative associations among girls. For the exposure measures in maternal blood, sex did not significantly modify the associations of exposure with the masculine score. For the femininity score (Table 4), we observed a similar consistent overall pattern of positive associations of exposures with the feminine score among boys and of negative associations with the feminine score among girls; the associations were significant for boys, but marginally significant for girls. The modification of exposure–outcome associations by sex was significant for the exposure matrix maternal milk and marginally significant for the maternal blood matrix. For both the masculine and the feminine scores, the matrix difference in terms of the strength of associations favoring the milk matrix is interesting and will be discussed later. For the PSAI difference score (feminine score – masculine score), no significant or marginally significant associations with exposure were seen. Complete results are given in Supplemental Material, Table S4.

**Table 4:** Results of multiple regression analyses of associations between POPs exposures measured in maternal blood or maternal milk and PSAI scores.

Exposure variable	Maternal blood, boys		Maternal blood, girls		Sex × exposure *p-*value	Maternal milk, boys		Maternal milk, girls		Sex × exposure *p-*value
	β (95% CI)	*p-*Value	β (95% CI)	*p-*Value		β (95% CI)	*p-*Value	β (95% CI)	*p-*Value	
Masculine score^*a*^
PCDD/F^*b*^	2.82 (–0.36, 6.01)	0.08	–1.89 (–3.94, 0.15)	0.07	0.20	3.18 (0.41, 6.77)	0.08	–3.40 (–6.03, –0.78)	0.01	0.01
PCB^*b*^	1.42 (–1.13, 3.96)	0.27	–0.88 (–2.59, 0.84)	0.32	0.31	1.95 (–1.08, 4.99)	0.21	–1.24 (–3.40, 0.91)	0.26	0.07
PCDD/F + PCB^*b*^	2.48 (–0.58, 5.55)	0.11	–1.72 (–3.81, 0.37)	0.11	0.19	2.88 (–0.59, 6.35)	0.10	–2.59 (–5.16, –0.02)	0.05	0.02
Feminine score^*a*^
PCDD/F^*b*^	2.60 (0.39, 4.80)	0.02	–1.74 (–4.22, 0.73)	0.17	0.09	4.02 (1.77, 6.28)	< 0.01	–2.88 (–6.11, 0.36)	0.08	< 0.01
PCB^*b*^	2.07 (0.35, 3.80)	0.02	–1.96 (–4.00, 0.08)	0.06	0.08	3.24 (1.35, 5.14)	< 0.01	–3.59 (–6.08, –1.10)	0.01	< 0.01
PCDD/F + PCB^*b*^	2.62 (0.52, 4.71)	0.02	–2.20 (–4.69, 0.29)	0.08	0.06	3.90 (1.74, 6.06)	< 0.01	–4.22 (–7.24, –1.19)	0.01	< 0.01
^***a***^Analyses of masculine scores included 52 boys and 56 girls for exposures measured in maternal blood (108 total), and 43 boys and 48 girls for exposures measured in maternal milk (91 total). Analyses of feminine scores included 52 boys and 57 girls for exposures measured in maternal blood (109 total), and 43 boys and 48 girls for exposures measured in maternal milk (91 total). ^***b***^Log_2_-transformed WHO_2005_-TEQ–weighted sum of PCDD/Fs, PCBs, or PCDD/Fs + PCBs measured in maternal blood lipid or breast milk lipid. Estimates of association for boys and girls were derived from separate models adjusted for age at examination, younger siblings (yes/no), older siblings (yes/no), birth weight, smoking during pregnancy (yes/no), alcohol consumption during pregnancy (any/none), German or non-German nationality, maternal IQ (Vocabulary subtest from German WAIS), and total maternal milk intake via nursing. Adjusted regression coefficients indicate the estimated difference in the PSAI score associated with a doubling of exposure. Interaction *p*-values were derived from models that included an interaction term for sex × exposure, plus lower-order terms for sex and exposure and the other covariates listed above.										

**Figure 1:**
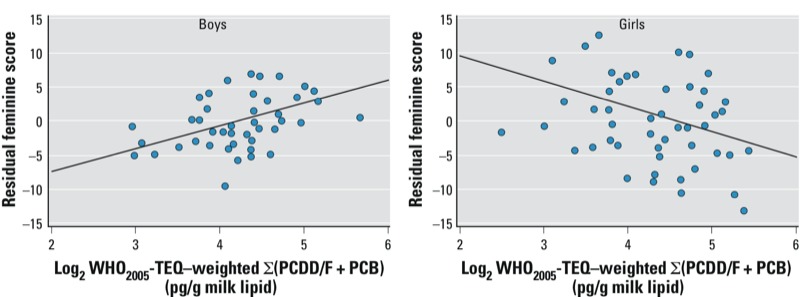
Partial regression plots of the PSAI feminine scores for ΣPCDD/F + PCB in maternal milk for boys and girls. Data points are the adjusted residuals of the multiple linear regression analyses where the ­exposure variable was left out (Table 4).

To add dose–response information besides inferential statistics, the association of prenatal POP exposure with PSAI ratings in both sexes is depicted in Figure 1. The partial regression plots show the inverse effects of prenatal PCDD/F + PCB exposure on the feminine score of boys and girls.

Among the set of covariates, a significant and interesting association with PSAI ratings appeared in the results of the multivariate regression analyses. The total postnatal maternal milk intake via nursing showed an almost identical pattern of associations with PSAI scores compared with prenatal POP exposure. For example, the feminine score increased by 2.1 for boys (*p* = 0.04) and decreased by 2.5 for girls (*p* = 0.04), adjusted for all other covariates and PCDD/F + PCB in maternal milk as a proxy of prenatal exposure, if the total milk consumption via breastfeeding increased by 100 L. This latter result of significant relations between sexually dimorphic behavior and total maternal milk intake via nursing is interesting, and will be discussed in “Discussion.”

## Discussion

Without additional information, causal inferences cannot be drawn from a single observational study with complex patterns of EDC exposure, such as this one. However, by placing our findings into a broader context of other epidemiological and experimental observations, a weight of the evidence (WOE) approach, based on some of the criteria developed to support causality in the context of smoking and lung cancer (Bradford Hill 1977), will be used to conclude whether the overall evidence for causal inferences is “sufficient,” “limited,” or “insufficient.” Such criteria, including temporality, dose response, internal and external consistency, strength of observed associations, and biological plausibility, have been used within narrative WOE approaches to come up with supporting causal inferences in the broader context of the impact of environmental EDCs on humans and wildlife (Bergman et al. 2013).

Using the PSAI, we found both positive and negative associations of prenatal exposure to different POP classes, namely PCDD/Fs and PCBs, with parent-reported behavioral sexual dimorphism in children at school age. This basically supports the findings of the Rotterdam study (Vreugdenhil et al. 2002), although some discrepancies of outcome between both studies must be considered. The consistent key finding in both studies is that PCBs and PCDD/Fs at environmental levels do exhibit pronounced associations with sexually dimorphic patterns of behavior in school-age children, based mostly on maternal reports of preferred toys, play activities, and sex-typical characteristics.

Although we found strong and consistent associations in the present study for the femininity PSAI scale, we also observed a consistent, although mostly not formally significant pattern of associations for the masculinity PSAI scale: positive associations for boys and negative ones for girls. These findings are only partly consistent with those of the previous study of Dutch children, which reported that prenatal exposure to PCBs was associated with significantly lower masculinity scores in boys and insignificantly higher masculinity scores in girls, whereas dioxin exposures were associated with higher femininity scores in both boys and girls (Vreugdenhil et al. 2002). More recently, and also using the PSAI, prenatal exposure to phthalates was reported to be associated with reduced masculine play in boys (Swan et al. 2010).

*Exposure*. Environmental levels of internal exposure to PCDD/Fs and PCBs were low relative to what has been reported in comparable studies. The average lipid-based concentration of ΣPCDD (WHO_1998_-TEQ) measured in breast milk samples from mothers in the Dutch cohort was 36.3 ng/kg (Vreugdenhil et al. 2002), whereas in this Duisburg study 10 years later, the corresponding value for ΣPCDD/F (WHO_2005_-TEQ, lipid based) was 11.6 pg/g (Table 2). Similarly, the average ΣPCDD + PCB (WHO_1998_-TEQ) was 68.1 ng/kg in Rotterdam, compared with 20.5 pg/g for our study (Table 2). As for indicator PCBs, the median value of unweighted ΣPCB (PCBs 138, 153, 180) measured in breast milk samples collected from 1993 through 1995 in a study of 171 mother–child pairs conducted in Düsseldorf was 405 ng/g milk lipids (Walkowiak et al. 2001), compared with 172 ng/g milk lipids in the Duisburg cohort (Wilhelm et al. 2008b). Thus, the levels of dioxins and PCBs were about 2–3 times higher in the early 1990s than in the present study.

*Outcome*. Neurodevelopmental deficit has been regarded as a particularly vulnerable end point of environmental exposure to PCBs (Schantz et al. 2003). Although such a deficit was not observed at the low levels of exposure in the Duisburg study (Wilhelm et al. 2008a), the findings of the present study suggest that environmental PCB exposures may affect behavioral sexual dimorphism, as evidenced by modification of play behaviors, toy preferences, and sex-typical characteristics as reported by their mothers or parents.

Vreugdenhil et al. (2002) also reported that prenatal exposures to PCBs and dioxins were associated with sex-specific behaviors, proposing a causative interpretation. However, whereas the Dutch study reported stronger associations between exposures and the masculine scale of the PSAI, we found stronger associations with the feminine scores. In addition, the Dutch study reported statistically significant PCB-related associations for boys only, whereas we estimated significant associations for both sexes. Finally, except for positive associations with femininity scores in boys, associations in boys and girls were in the opposite direction between the two studies. We do not have an explanation for these discrepancies.

With increasing POP concentrations, we found more behavioral femininity in boys and less femininity in girls, but also more masculinity in boys and less masculinity in girls. This apparent contradiction might reflect a bi- or even multidimensional factorial structure of behavioral sexual dimorphism (Meyer-Bahlburg et al. 1994) rather than a one-dimensional male–female continuum.

PCDD/Fs and PCBs measured in breast milk collected within the first 3 weeks following birth were more strongly associated with sexually dimorphic outcomes than exposures measured in maternal blood collected between weeks 28 and 43 (median, 32 weeks). A similar matrix discrepancy was also reported for the association between dioxins/PCBs and serum dehydroepiandrosterone concentrations (an indicator of adrenal maturation) in the Duisburg cohort (Rennert et al. 2012).

Although the primary focus of this study was on links between *in utero* exposure to PCBs and PCDD/Fs and sexually dimorphic behavior, we also found significant associations between the femininity PSAI scores and total postnatal milk intake as a covariate in the regression model. This may be taken as suggestive evidence for additional postnatal modification of behavioral sex dimorphism, although further support for this observation using more adequate measures of postnatal POP exposure is needed.

*Endocrine disruption*. Among the many endocrine-mediated features of sexual development, the clearest evidence for hormonal influences on human behavioral development comes from studies of childhood play (Collaer and Hines 1995). The characterization of dioxins and PCBs and/or their metabolites as EDCs is consistent with the outcome of this study. The different associations observed for the masculine PSAI score [(anti)androgenicity] and for the feminine PSAI score [(anti)estrogenicity], as given in Table 4, can be interpreted as presumably reflecting differential hormonal activities of various congener groups within the complex POP mixtures.

This endocrine hypothesis is supported partly by evidence from experiments in intact animals. Sex hormones and P450 enzymes (i.e., aromatase) involved in the conversion of androgens to estrogens were shown to be affected by PCBs after prenatal exposure (Hany et al. 1999; Kaya et al. 2002). Female offspring of rats with dietary exposure to a PCB mixture during gestation exhibited increased uterus weight as well as dose-dependent decreases in testosterone and estradiol (Kaya et al. 2002). In male pups, prenatal exposure to a PCB mixture was associated with reduced brain aromatase activity in the hypothalamic preoptic area and, at an older postnatal age, with increased feminized behavior (increased sweet preference) as well as lower testis weights and reduced testosterone levels that persisted into adulthood (Hany et al. 1999). Another study that used the sweet preference model reported that maternal and lactational exposure to a TCDD (tetrachlorodibenzo-*p*-dioxin) congener (2,3,7,8-TCDD) or to coplanar PCBs (77 and 126) was associated with evidence of masculinization in female rats, suggesting antiestrogenic activity (Amin et al. 2000).

Apart from this experimental evidence there is also recent support based on human data that dioxins and dioxin-like POPs may contribute to endocrine-mediated sexually dimorphic outcomes. Using anogenital distance as the end point, which is known to be longer in males than in females, significant negative associations were reported between *in utero* levels of dioxin-like compounds and ADG in newborn boys but not girls; these were still negative at an older age (mean, 16 months) but no longer significant (Vafeiadi et al. 2013). However, our observations rather support the possibility that EDC-linked behavioral sexual dimorphism may be lasting at least until school age.

*Strengths and limitations*. A remarkable feature of this study relates to the fact that robust associations were found despite its small sample size. This indicates what is called “strength of effect” in the Bradford Hill framework. Also, the prospective nature of the study design with measures of exposure preceding the assessment of behavioral sexual dimorphism fulfills the criterion of “temporality.” Additionally, by demonstrating linear dose–response relationships (Figure 1) a third important criterion within the Bradford Hill framework can be considered as given.

However, some limitations of the study must be also mentioned. The PSAI was developed for use in preschool children, whereas most of the children in our cohort were > 6 years of age (Table 1). Also, as used in the present study, the PSAI does not fully reflect behavioral aspects of sexual dimorphism in children’s play behavior today, because computer games and smartphone use are not considered. Thus, our observations are valid only within the constraints of the sex-typed activities and toy preferences sampled in our PSAI version. Furthermore, although the psychometric characteristics of the PSAI are satisfactory (Golombok and Rust 1993), they have been established only for the composite score. Our sample was small, and loss to follow-up resulted in a more middle- to upper-class composition compared the study cohort at first recruitment. Therefore, care must be taken to generalize our findings to the general population. Because of ethical requirements the parents received feedback of results at the earliest possible convenience. Thus, written information about individual exposure and neurodevelopmental outcome was given as early as possible in 2005. POP concentrations of WHO_2005_-TEQ in blood and milk were given. However, because no reference values exist, no qualifying statement was possible, other than for three single indicator PCBs (PCBs 138, 153, 180). For just one child in the initial Duisburg cohort, German reference values (population-based 95th percentiles) for these three compounds were exceeded. It is unlikely, therefore, that the formal violation of the double-blind principle may have biased the study outcome. This interpretation is supported by the fact that the modification of the masculine score was less pronounced than that of the feminine score.

## Conclusions

This study provides additional evidence that low levels of prenatal environmental exposure to dioxins and PCBs modify behavioral sexual dimorphism in school-age children. Linear dose–response relationships were established between exposure and outcome (PSAI ratings). Specifically, exposure to dioxins and PCBs in boys was associated with more feminine behavior, whereas in girls exposure was associated with less feminine behavior. However, consistent but less pronounced associations were also found between exposure and rated masculinity, positive for boys and negative for girls. These POP complexes have been shown to have endocrine-disrupting properties in a variety of biological models. They have also been found to be linked with sexually dimorphic behavior in the Dutch study (Vreugdenhil et al. 2002), and were previously reported to be associated with altered cord serum estradiol and testosterone levels in the Duisburg study cohort (Cao et al. 2008). Thus, based on the Bradford Hill criteria of causality, we conclude that the overall evidence that PCBs and dioxins modify sexually dimorphic behavior in children is “sufficient.” From what is known about the neurobiology of sex dimorphism during brain development, we believe that this modification is probably based on exposure-induced imbalances within the HPG axis. However, uncertainties remain: First, the discrepant findings for the masculine and feminine PSAI scales must be clarified. Second, due to a lack of adequate markers of postnatal POP exposure, such as those used in a previous PCB study (Walkowiak et al. 2001), our observation of associations between postnatal exposure via breastfeeding and sexually dimorphic behavior in children is still of only “limited” evidence. More focused research is needed to study whether postnatal POP exposure may also have an impact on behavioral sexual dimorphism. Third, more research is also needed on the issue of a possible endocrine relevance of other environmental EDCs for this end point. Additional research is also important to clarify whether these findings extend beyond puberty.

## Supplemental Material

(315 KB) PDF
