# Supplementary material for: Behavioral Sexual Dimorphism in School-Age Children and Early Developmental Exposure to Dioxins and PCBs: A Follow-Up Study of the Duisburg Cohort
Source: Environ Health Perspect. 2013 Nov 22;122(3):292–8. doi: 10.1289/ehp.1306533 (PMC3948031; doi:10.1289/ehp.1306533)
Supplement: (315 KB) PDF [file ehp.1306533.s001.508.pdf]

Supplemental Material

Behavioral Sexual Dimorphism in School-Age Children and Early Developmental Exposure to Dioxins and PCBs: A Follow-Up Study of the Duisburg Cohort

Gerhard Winneke, Ulrich Ranft, Jürgen Wittsiepe, Monika Kasper-Sonnenberg, Peter Fürst, Ursula Krämer, Gabriele Seitner, and Michael Wilhelm

Table of Contents

Table S1: Matrix of correlations of POP concentrations within and between blood/milk matrices.

Table S2: Concentrations of PCBs, PCDDs and PCDFs measured in blood and milk lipids.

Table S3: Description of pre- and postnatal exposure to POPs in the Duisburg birth cohort.

Table S4: Results of multiple regression analyses of associations between POP exposures and PSAI difference score.

Table S1: Matrix of correlations of POP concentrations within and between blood/milk matrices.

	Blood: PCDD/F	Blood: non-ortho PCB	Blood: mono-ortho PCB	Blood: PCB	Blood: Σ PCB	Blood: PCDD/F + PCB	
Milk: PCDD/F	0.90	0.77	0.88	0.80	0.78	0.97	Blood: PCDD/F
Milk: non-ortho PCB	0.82	0.84	0.86	1.00	0.72	0.89	Blood: non-ortho PCB
Milk: mono-ortho PCB	0.91	0.90	0.94	0.89	0.92	0.93	Blood: mono-ortho PCB
Milk: PCB	0.83	1.00	0.92	0.87	0.76	0.91	Blood: PCB
Milk: Σ PCB	0.80	0.81	0.92	0.83	0.94	0.82	Blood: Σ PCB
Milk: PCDD/F + PCB	0.95	0.95	0.96	0.96	0.87	0.91	Blood: PCDD/F + PCB
	Milk: PCDD/F	Milk: non-ortho PCB	Milk: mono-ortho PCB	Milk: PCB	Milk: Σ PCB	Milk: PCDD/F + PCB	

Correlations are based on 101 milk samples, 118 blood samples, and 98 paired milk X blood samples.

PCDD/F = WHO_{2005} TEq-weighted summary measures; Σ PCB = sum of PCB concentrations of 6 marker congeners (#28, #52, #101, #138, #153, #180).

Table S2: PCDDs, PCDFs and PCBs measured in blood and milk lipids. Toxic equivalent factors (TEq) according to WHO_{2005} are assigned to each compound as indicators of Ah-receptor mediated toxicity relative to # D48).

**Compounds**	WHO_{2005} TEq values
**PCDDs**	
2,3,7,8-TetraCDD (# D48)	1
1,2,3,7,8-PentaCDD (# D54)	1
1,2,3,4,7,8-HexaCDD (# D66)	0.1
1,2,3,6,7,8-HexaCDD (# D67)	0.1
1,2,3,7,8,9-HexaCDD (# D70)	0.1
1,2,3,4,6,7,8-HeptaCDD (# D73)	0.01
OctaCDD (# D75)	0.0003
**PCDFs**	
2,3,7,8-TetraCDF (# F83)	0.1
1,2,3,7,8-PentaCDF (# F94)	0.03
2,3,4,7,8-PentaCDF (# F114)	0.3
1,2,3,4,7,8-HexaCDF (# F118)	0.1
1,2,3,6,7,8-HexaCDF (# F121)	0.1
1,2,3,7,8,9-HexaCDF (# F124)	0.1
2,3,4,6,7,8-HexaCDF (# F130)	0.1
1,2,3,4,6,7,8-HeptaCDF (# F131)	0.01
1,2,3,4,7,8,9-HeptaCDF (# F134)	0.01
OctaCDF (# F135)	0.0003
**non-ortho-substituted PCBs**	
3,3',4,4'-TetraCB (# 77)	0.0001
3,4,4',5-TetraCB (# 81)	0.0003
3,3',4,4',5-PentaCB (# 126)	0.1
3,3',4,4',5,5'-HexaCB (# 169)	0.03
**mono-ortho-substituted PCBs**	
2,3,3',4,4'-PentaCB (# 105)	0.00003
2,3,4,4',5-PentaCB (# 114)	0.00003
2,3',4,4',5-PentaCB (# 118)	0.00003
2',3,4,4',5-PentaCB (# 123)	0.00003
2,3,3',4,4',5-HexaCB (# 156)	0.00003
2,3,3',4,4',5'-HexaCB (# 157)	0.00003
2,3',4,4',5,5'-HexaCB (# 167)	0.00003
2,3,3',4,4',5,5'-HeptaCB (# 189)	0.00003
**non dioxin-like PCBs**	
2,4,4’-TriCB (# 28)	na
2,2’,5,5’-TetraCB (# 52)	na
2,2’,4,5,5’-PentaCB (# 101)	na
2,2’,3’,4,4’,5-HexaCB (# 138)	na
2,2’,4,4’,5,5’-HexaCB (# 153)	na
2,2’,3,4,4’,5,5’-HeptaCB (# 180)	na

na: not applicable

Table S3: Description of prenatal exposure to polychlorinated POPs in the Duisburg birth cohort.

Polychlorinated POPs in maternal blood and milk	N	Mean ± SD	Min	P05	P50	P95	Max
Non-ortho PCB [pg/g blood lipid] [a]	118	6.1 ± 3.5	1.1	1.8	5.4	12.2	23.5
mono-ortho PCB[pg/g blood lipid] [a]	118	0.8 ± 0.4	0.1	0.2	0.7	1.7	2.0
Σ PCB [µg/g blood lipid] [b]	118	189.2 ± 175.8	18.4	63.8	157.7	371.4	1813.8
Non-ortho PCB [pg/g milk lipid] [a]	101	8.2 ± 4.6	1.8	2.9	7.9	15.0	30.4
mono-ortho PCB [pg/g milk lipid] [a]	101	0.8 ± 0.3	0.1	0.3	0.7	1.3	1.8
Σ PCB [µg/g milk lipid] [b]	101	201.2 ± 159.9	23.5	86.5	171.5	377.5	1530.5

N: sample size; Mean: arithmetic mean; SD: standard deviation; Min: minimum; P05, P50 and P95: 5th, 50th and 95th percentile; Max: maximum

[a] WHO-TEq-weighted sum

[b] Sum of non-dioxin-like PCBs (#28, #52, #101, #138, #153 and #180)

Table S4: Results of multiple regression analyses of associations between POP exposures and PSAI difference score.

Exposure variable[a]	Maternal blood, Boys		Maternal blood, Girls		Sex × Expo	Maternal milk, Boys		Maternal milk, Girls		Sex × Expo
	β (95% CI)	p	β (95% CI)	p	p	β (95% CI)	p	β (95% CI)	p	p
PCDD/F [a]	-0.23 (-4.33, 3.88)	0.91	-0.24 (-3.16, 2.67)	0.87	0.52	0.84 (-3.45, 5.13)	0.70	-0.25 (-4.08, 3.58)	0.90	0.67
PCB [b]	0.66 (-2.55, 3.87)	0.69	-1.33 (-3.70, 1.04)	0.27	0.23	1.29 (-2.27, 4.85)	0.48	-2.51 (-5.42, 0.40)	0.09	0.23
PCDD/F+PCB [b]	0.13 (-3.80, 4.06)	0.95	-0.90 (-3.84, 2.05)	0.55	0.16	1.02 (-3.11, 5.15)	0.63	-2.12 (-5.74, 1.49)	0.25	0.39

Analyses of PSAI difference scores included 52 boys and 56 girls for exposures measured in maternal blood (108 total), and 43 boys and 48 girls for exposures measured in maternal blood (91 total).

[a]Log_2-transformed WHO_{2005}-TEq-weighted sum of PCDD/F, PCB or PCDD/F+PCB measured in maternal blood lipid or breast milk lipid, respectively. Estimates of association for boys and girls were derived from separate models adjusted for age at examination, younger siblings (yes/no), older siblings (yes/no), birth weight, smoking during pregnancy (yes/no), alcohol consumption during pregnancy (any/none), German or non-German nationality, maternal IQ (vocabulary subtest from WAIS), and total maternal milk intake via nursing. Adjusted regression coefficients indicate the estimated difference in the PSAI score associated with a doubling of exposure. Interaction p-values were derived from models that included an interaction term for sex*exposure, plus lower order terms for sex and exposure and the other covariates listed above.
